# Impact of C-reactive protein on the effect of Roxadustat for the treatment of anemia in chronic kidney disease: a systematic review of randomized controlled trials

**DOI:** 10.1186/s12882-024-03474-5

**Published:** 2024-02-05

**Authors:** Xiaoyu Luo, Guoli Li, Hongyu Yang, Lang Chen, Yinyan Gao, Jing Cong, Hui Luo, Weiru Zhang

**Affiliations:** 1 Department of Rheumatology and Immunology, Xiangya Hospital, Central South University, Changsha, 410008 Hunan People’s Republic of China; 2 National Clinical Research Center for Geriatric Disorders (Xiangya Hospital), Changsha, 410008 Hunan People’s Republic of China; 3 Department of Nephrology, Hunan Clinical Research Center for Chronic Kidney Disease, Hunan Provincial People’s Hospital, The First Affiliated Hospital of Hunan Normal University, Changsha, 410005 Hunan People’s Republic of China; 4 Department of Hepatopancreatobiliary Surgery, The Third Xiangya Hospital, Central South University, Changsha, 410008 Hunan People’s Republic of China; 5 Department of Epidemiology and Biostatistics, Xiangya School of Public Health, Central South University, Changsha, 410008 Hunan People’s Republic of China; 6 Department of General Medicine, Xiangya Hospital, Central South University, Changsha, 410008 Hunan People’s Republic of China; 7 National Medical Metabolomics International Collaborative Research Center, Xiangya Hospital, Central South University, Changsha, 410008 People’s Republic of China

**Keywords:** C-reactive protein, Anemia, CKD, Systematic review, Roxadustat, Clinical trials

## Abstract

**Background:**

Chronic inflammation, reflected by an increased blood C-reactive protein (CRP) level, is common in patients with chronic kidney disease (CKD) and is involved in the development of renal anemia. This systematic review aims to investigate the impacts of CRP on the efficacy of hypoxia-inducible factor-prolyl hydroxylase inhibitors (HIF-PHIs) in the treatment of renal anemia in patients with CKD.

**Methods:**

We conducted a comprehensive search of electronic databases including Pubmed, Web of Science, Embase, Cochrane Library, CNKI, Wanfang, and the International Clinical Trials Registry Platform (ICTRP), from their inception to May 19, 2022. We systematically reviewed evidence from randomized controlled trials using HIF-PHIs for renal anemia treatment. The mean difference (MD) in changes in hemoglobin concentration (∆Hb) before and after treatment served as the meta-analysis outcome, utilizing a random-effects model. We compared groups with CRP levels greater than or equal to the upper limit of normal (ULN) and less than the ULN. Additionally, further analysis was conducted in the CRP ≥ ULN group comparing HIF-PHIs and erythropoiesis-stimulating agents (ESA).

**Results:**

A total of 7 studies from 6 publications were included in the analysis. In the comparison between the CRP ≥ ULN group and the CRP < ULN group, 524 patients from 4 studies were incorporated into the analysis. All patients received roxadustat as the primary intervention. The pooled results revealed no significant difference in ΔHb between patients with CRP ≥ ULN and CRP < ULN at baseline (Mean Difference: 0.00, 95% Confidence Interval: -0.32 to 0.33, *P* = 0.99). Moreover, within the CRP ≥ ULN group, three studies involving 1399 patients compared the efficacy of roxadustat and erythropoiesis-stimulating agents (ESAs). The results indicated no significant difference in ΔHb between patients treated with ESAs and HIF-PHIs (Mean Difference: 0.24, 95% Confidence Interval: -0.08 to 0.56, *P* = 0.14). In terms of medication dosage, an increase in ESA dose over time was observed across various studies, particularly evident in the CRP ≥ ULN group, while the dose of roxadustat remains constant over time and is not influenced by the baseline levels of CRP.

**Conclusions:**

Our systematic review demonstrates that roxadustat exhibits similar efficacy across different CRP levels. Moreover, within the CRP ≥ ULN group, roxadustat can maintain efficacy comparable to ESA without the necessity for dose escalation.

**Trial registration:**

CRD42023396704.

**Supplementary Information:**

The online version contains supplementary material available at 10.1186/s12882-024-03474-5.

## Background

Chronic kidney disease(CKD) is a progressive condition resulting from a heterogeneous range of disease, ultimately leading to irreversible alterations in kidney function and structure [1]. Globally, over 10% of the population is affected by CKD [2], making it an increasingly significant health burden on society. While the precise pathological mechanism driving CKD are not fully understood, a growing list of primary and secondary risk factors has been identified. Among these factors, anemia emerges as a hallmark complication of CKD and is associated with a poor prognosis. Its occurrence is linked to various elements such as impaired renal endocrine function, resulting in inadequate secretion of erythropoietin (EPO), urotoxic toxin accumulation, chronic inflammation, impaired iron metabolism, and a shortened half-life of erythrocytes [3]. Consequently, erythropoiesis-stimulating agents (ESA) have been the standard therapy for treatment renal anemia since the 1990s.

Simultaneously, chronic inflammation is common in CKD patients [3, 4]. As a marker of systemic inflammation, C-reactive protein (CRP) also stands out as an independent risk factor for CKD [5–7]. Previous studies have indicated that ESA is less effective in patients with renal anemia with high CRP levels [8, 9]. The necessity for increased dose required to maintain target hemoglobin concentration may elevate the risk of cardiovascular events and tumorigenesis [10]. These findings suggest that CRP levels may impact the efficacy of drugs used for treating CKD anemia.

Hypoxia-inducible factor-prolyl hydroxylase inhibitors (HIF-PHIs) represent a novel class of drugs for treating anemia in CKD. These drugs stabilize hypoxia-inducible factor (HIF) by inhibiting hypoxia-inducible factor prolyl hydroxylase. Through intermittent administration, HIF-PHIs simulate transient physiological hypoxia, thereby stimulating endogenous EPO synthesis. In comparison to conventional ESA, HIF-PHIs offer the advantage of avoiding side effects associated with high EPO concentration. Presently, six HIF-PHIs (roxadustat, daprodustat, desidustat, enarodustat, molidustat, and vadadustat) have been developed for global clinical use. Their safety and efficacy have been demonstrated in CKD, encompassing both dialysis-dependent and non-dependent patients [11–14]. Notably, the impact of CRP on the outcomes has been explored in several large randomized-controlled trials (RCT) of HIF-PHIs for treating renal anemia [15–20]. However, these reports were with variable findings, leaving the question of whether CRP levels affect HIF-PHIs efficacy unclear. To address this question, our study systematically reviewed RCT of HIF-PHIs, aiming to provide clarity on this aspect.

## Methods

This study adhered to the Preferred Reporting Items for Systematic Evaluation and Meta-Analysis (PRISMA) [21]. Literature selection, risk of bias assessment, certainty of evidence evaluation, and data extraction were independently conducted by two investigators, XYL and GLL, with any disagreements resolved through consultation with a third investigator, HYY. Our research was prospectively registered in the PROSPERO database with the registration number CRD42023396704.

### Search strategy

We systematically searched Pubmed, Web of Science, Embase, Cochrane Library, CNKI, and Wanfang databases from their inception to May 19, 2022 to identify eligible studies. Search keywords included "renal insufficiency, chronic", "chronic kidney diseases", "chronic renal insufficiencies", "hypoxia-inducible factor-prolyl hydroxylase inhibitors", "HIF- PHI", etc. The filter for identifying RCT studies followed to the recommendations of the Harvard Library Research Guide [22], and a highly sensitive search strategy was employed to comprehensively detect relevant studies. Additionally, the International Clinical Trials Registry Platform (ICTRP), a global repository of registered clinical studies worldwide, was searched for registered studies with reported results. The complete search strategy is available in the Supplementary Material.

### Inclusion and exclusion criteria

Original studies were included if they met the following criteria: (1)RCT design; (2)Use of HIF-PHIs as interventions, with or without prior ESA treatment; (3)Conducted in patients with CKD, with or without dialysis treatment; (4)Reporting outcomes related to the impact of CRP levels on the efficacy of HIF-PHIs, such as changes in hemoglobin concentration (∆Hb) from baseline to the end-of-treatment(EoT). Studies lacking qualifying controls or relevant data on drug effectiveness were excluded. Publications without primary data, such as reviews, comments, and protocols were also excluded. Conference abstracts were omitted due to their lack of peer review. Non-English non-Chinese literature was reviewed using translation software, and professional translators were consulted when necessary.

### Risk of bias and certainty of evidence assessment

The study’s risk of bias was evaluated using version 2 of the risk-of-bias assessment tool provided by the Cochrane Collaboration Network [23]. Bias assessment covered seven domains, encompassing random sequence generation, allocation concealment, blinding of participants and personnel, blinding of outcome assessment, incomplete outcome data, selective reporting, and other biases.

Certainty of evidence for each outcome indicator was assessed utilizing the Grading of Recommendations Assessment, Development, and Evaluation (GRADE) framework. The assessment of study quality included considerations such as study design, inconsistency, indirectness, imprecision, and other relevant factors. Based on this quality assessment, evidence was categorized into high quality, moderate quality, low quality, and very low quality.

### Data extraction and analysis

Data extraction employed a pre-developed form, encompassing study design, patient characteristics, the number of patients available for analysis, demographic information, treatment background, interventions, CRP type and upper limit of normal value, study registration number, and outcome data for analysis. Efficacy was evaluated using mean differences(MD) and 95% confidence intervals(CI). A random-effects model was used for data combination, with a fixed-effects model to assess model robustness and sensitivity to anomalous outcomes. Results are visually presented in forest plots. Heterogeneity was evaluated using the chi-square test and quantitative analysis utilized the I^2^ test. The significance of I^2^ statistics was considered with regard to both the direction of the outcome effect and the strength of the evidence of heterogeneity. Subgroup analyses were conducted based on pre-determined factors related to the study design and outcome reporting, including the type of HIF-PHI used, dialysis dependence, prior ESA treatment, and the availability of publicly reported results. Sensitivity analysis involved individual study exclusion and subsequent reanalysis of the remaining studies. Funnel plots were employed to assess publication bias in the literature. Cochrane's Review Manager (RevMan) version 5.4 and R Language 4.0.5 were utilized for meta-analyses.

## Results

### Study characteristics

**Fig. 1:**
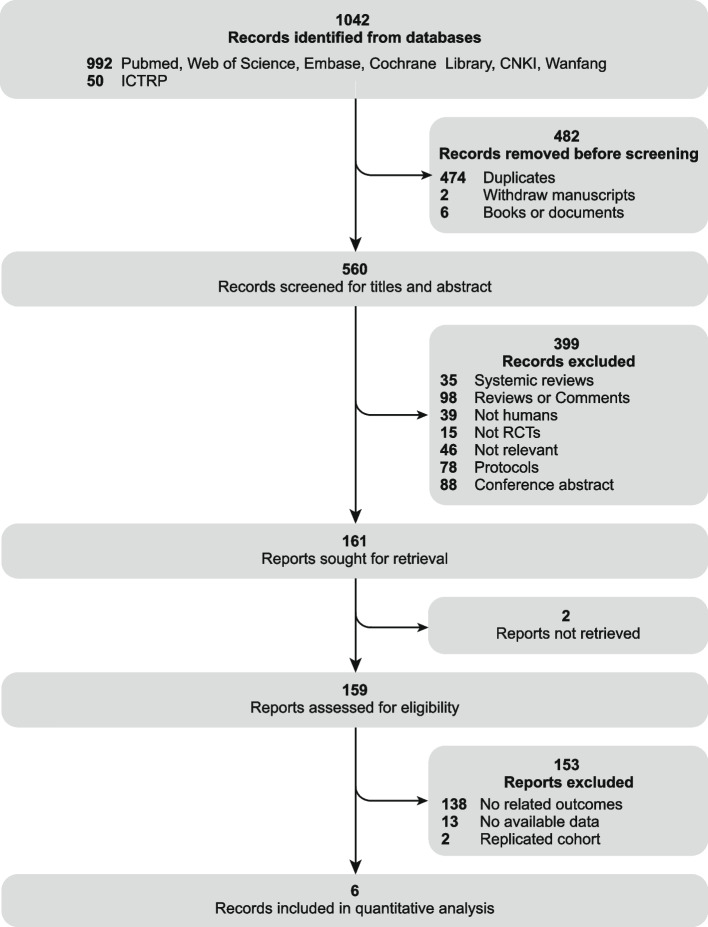
PRISMA 2020 flow diagram. ICTRP, International Clinical Trials Registry Platform; RCT, randomized-controlled trials

Following the systematic search of PubMed, Web of Science, Embase, Cochrane Library, CNKI, and Wanfang databases, a total of 992 publications were identified. Through the search in ICTRP, 50 relevant trials with reported results were retrieved. A total of 482 records were excluded before review due to reasons such as duplication, withdrawal, and classification as books or documents. Upon reviewing the titles and abstracts of the records of remaining 560 records, 399 were subsequently excluded as they did not meet the pre-determined criteria. A comprehensive review of the full text of 161 eligible publications led to the inclusion of 6 publications representing 7 studies [15–20]. In the comparison between the CRP ≥ ULN group and the CRP < ULN group, we included 524 patients from 4 studies in our analysis. Within the CRP ≥ ULN group, three studies encompassing 1399 patients were conducted to assess the efficacy of roxadustat compared to erythropoiesis-stimulating agents (ESAs). Notably, the publication by Akizawa et al*.* (2019) [19] reported results from two independently registered and implemented studies, both independently included in the analysis. Furthermore, outcome data from Charytan et al*.* (2021) [17] were reported in official publications that could not be extracted; however, relevant disclosures were accessible on the clinical research registry website. Information about the study design and implementation was obtained from the publication, while outcomes data were retrieved from the registry website. Despite the absence of restrictions on the type of HIF-PHIs during the literature search and study availability assessment, all eventually enrolled studies employed roxadustat as the primary intervention. Therefore, the predetermined HIF-PHI drug classification could not be applied in the subsequent subgroup analysis. The inclusion and exclusion process are depicted in the PRISMA flow diagram Fig. 1, and characteristics of the included studies are summarized in Table 1.

**Table 1:** Characteristics of included studies

Study	Design			Patients	Relevant Patients	Male(%) ^a^	Mean Age ^a^	ESA Treatment	Interventions	Duration	Analyzed Patients	CRP Type	Baseline CRP/hsCRP	CRP ULN	TrailNo
	**Phase**	**Patients**	**Control**												
**Effect of CRP on the efficacy of HIF-PHIs**															
Provenzano et al. 2016 [20]	2	Open-labeled	Dosage-controlled	NDD-CKD	137	36.6	64.4 (25, 83)^b^	Naïve	Roxadustat	16-24w	137	CRP (mg/dl)	-	5	NCT01244763
Akizawa et al. 2019 [1]	3	Open-labeled	Noncomparative	HD-CKD	74	74.3	66.2 (12.1)^b^	Naïve	Roxadustat	24-52w	74	HsCRP (nmol/l)	27.313 (74.988) Mean (SD)	28.57	NCT02779764
Akizawa et al. 2019 [2]	3	Open-labeled	Noncomparative	HD-CKD	163	60.1	62.8 (11.8)^b^	Converted	Roxadustat	24-52w	163	HsCRP (nmol/l)	13.038 (22.043) Mean (SD)	28.57	NCT02780141
Akizawa et al. 2020 [18]	3	Double-blinded	Active-controlled	HD-CKD	150	67.3	64.6 (11.7)^b^	Converted	Roxadustat	24w	150	HsCRP (mg/l)	1.3246 (2.4124) Mean (SD)	3	NCT02952092
**Efficacy of HIF-PHIs in CRP ≥ ULN group compared with ESAs**															
Provenzano et al. 2021 [16]	3	Open-labeled	Active-controlled	ID-CKD	450	59.2	53.8 (14.7)^b^	Naïve	Roxadustat, Epoetin alfa	52w	450	hsCRP	-		NCT02052310
Charytan et al. 2021 [17]	3	Open-labeled	Active-controlled	DD-CKD	368	50.5	68.4% between 18–65 31.6% ≥ 65^c^	Converted	Roxadustat, Epoetin alfa	24w	368	HsCRP (mg/dl)	-	5	NCT02273726
Fishbane et al. 2022 [15]	3	Open-labeled	Active-controlled	DD-CKD	581	59.5	53.5 (15.3)^b^	Converted	Roxadustat, Epoetin alfa	52w	581	hsCRP (mg/dl)	Roxadustat 0.4 (0.2–1.1) Median (IQR) Epoetin alfa 0.4 (0.2–1.0)^d^	5	NCT02174731

*DD* dialysis-dependent, *NDD* non-dialysis-dependent, *ID* incident to dialysis, *CKD* chronic kidney disease, *CRP* c-reactive protein, *hs-CRP* hypersensitive-c-reactive protein, *ULN* upper limit of normal

^a^Male(%) and Mean Age is for those who receive Roxadustat treatment

^b^Mean ± standard deviation

^c^Percentage of age group

^d^Median CRP of CRP ≥ ULN group

### Risk of bias assessment

**Fig. 2:**
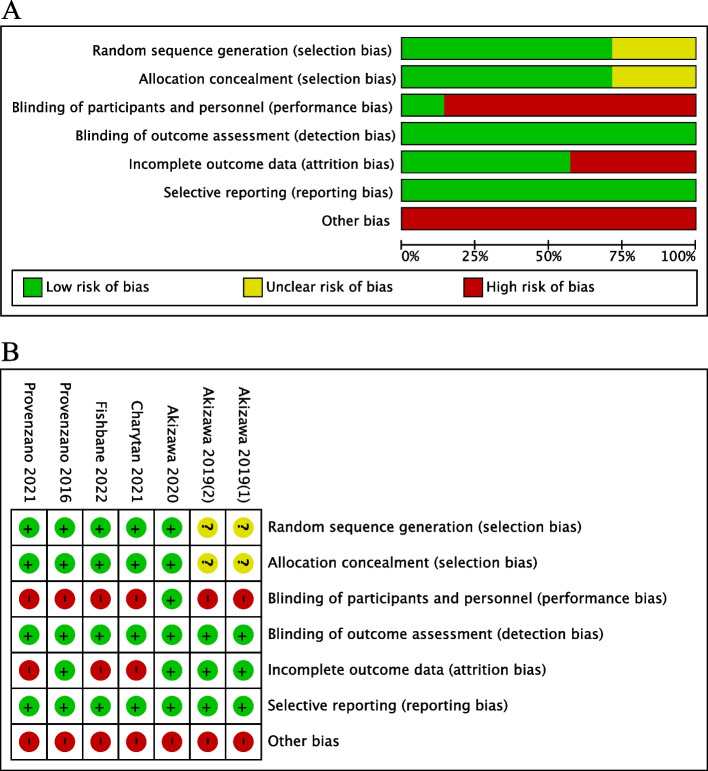
Risk of bias assessment. **A** Risk of bias graph: review authors’ judgments about each risk of bias item presented as percentages across all included studies. **B** Risk of bias summary: review authors’ judgments about each risk of bias item for each included study. The items are scored ( +) low risk; (-) high risk; (?) some concerns

Details of the bias assessment tools are presented in Fig. 2. The risk of bias was determined to be high across all studies, as they were enterprise-sponsored, resulting in a high risk of bias. Except for one study that ensured double-blinding of the intervention, most studies were open-label due to the use of ESAs as a control intervention. Variances in treatment implementation made maintaining blinding challenging. Studies by Fishbane et al*. *2022 [15], Provenzano et al*. *2021 [16], and Charytan et al*.* 2021 [17] were considered to have a high attrition bias because of the higher rate of loss of follow-up. The study by Akizawaet al*. *2019 [19] could not assess the risk of selection bias as it did not report the method of random sequence generation and allocation concealment.

### Meta-analysis

#### Effect of CRP levels on the efficacy of HIF-PHIs

##### Overall effect

**Fig. 3:**
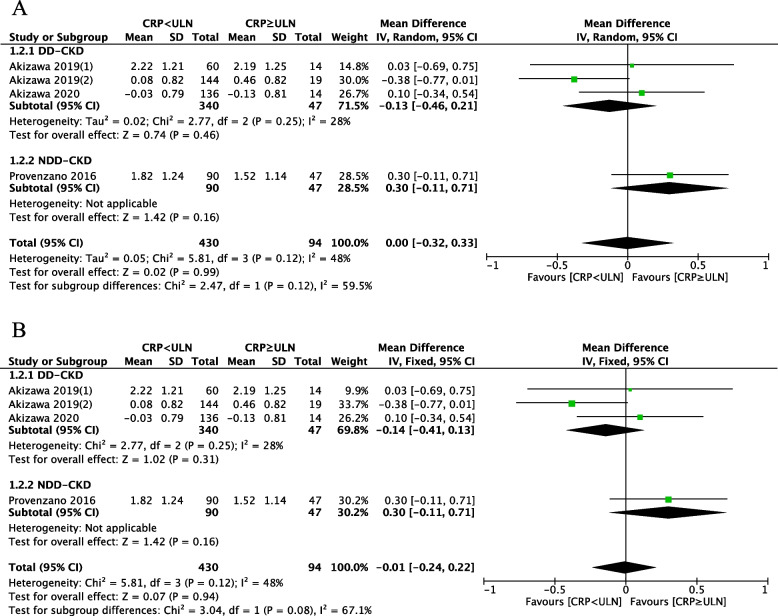
Forest plot of the effect of CRP levels on the efficacy of HIF-PHIs subgroup by dialysis-dependent type. **A** Random-effect model; **B** Fixed-effect model. CRP: c-reactive protein; ULN, upper limit of normal; Mean, mean change of Hb from baseline to end of treatment, g/dl; SD, Standard deviation; CI: confidence interval; Total: Total number of patients in the study group; DD-CKD: dialysis-dependent CKD, NDD-CKD: non-dialysis-dependent CKD; df: degrees of freedom; I^2^, I-squared

Four studies reported changes in hemoglobin from baseline to EoT after HIF-PHI treatment in patients categorized by CRP ≥ ULN and CRP < ULN at enrollment. No significant difference in ∆Hb between the two groups was observed, utilizing both a random-effects model (MD: 0.00, 95%CI: -0.32–0.33, *P* = 0.99) (Fig. 3A) and a fixed-effects model (MD: -0.01, 95%CI: -0.24–0.22, *P* = 0.94) (Fig. 3B). Overall heterogeneity was not significant (I^2^ = 48%, *P* = 0.12). Table 2 provides a summary of evidence quality based on the GRADE framework. Findings related to the effect of CRP levels on the efficacy of HIF-PHIs were considered low-quality evidence.

**Table 2:** Certainty of evidence and summary effect estimates assessed by GRADE (grading of recommendations, assessment, development, and evaluation) of the study outcomes

Outcomes	Summary of findings		Quality assessment					Certainty of evidence	Importance
	No. of participants (studies)	MD (95%CI)	Study design	Inconsistency	Indirectness	Imprecision	Other consideration		
ΔHb compared between CRP ≥ ULN and CRP	524 (4 RCTs)	MD 0.00 [-0.32,0.33]	Very serious ^a, b2^	not serious	not serious	not serious	none	⨁⨁◯◯ Low	CRITICAL
ΔHb in CRP ≥ ULN compared between HIF-PHI and control	1399 (3 RCTs)	MD 0.24 [-0.08,0.56]	Very serious ^b, c, d^	very serious ^e^	not serious	not serious	none	⨁◯◯◯ Very low	CRITICAL

*MD* mean difference, *CI* confidence interval

GRADE Working Group grades of evidence

High quality: very confident that the true effect lies close to that of the estimate of the effect

Moderate quality: moderately confident in the effect estimate, and the true effect is likely to be close to the estimate of the effect, but there is a possibility that it is substantially different

Low quality: confidence in the effect estimate is limited, and the true effect could be substantially different from the estimate of the effect

Very low quality: very little confidence in the effect estimate, and the true effect is likely to be substantially different from the estimate of effect

Explanations

^a^Three of the four studies were open-label

^b^All studies are sponsored by the enterprise

^c^All studies are open-label

^d^All studies have a high rate of loss of follow-up

^e^The I2 statistic is considerable and adjustment according to previous ES A treatment still failed to resolve inter- and intra-group heterogeneity

#### Subgroup analysis

Results of subgroup analysis based on dialysis dependence are presented in Fig. 3. The analysis includes three studies in the dialysis-dependent CKD (DD-CKD) group and one study in the non-dialysis-dependent CKD (NDD-CKD) group. In Fig. 3A, the random-effects model analysis revealed no significant difference in ∆Hb between patients with baseline CRP ≥ ULN and CRP < ULN, both within the DD-CKD group and the NDD-CKD group (DD-CKD group: MD: -0.13, 95%CI: -0.46–0.21, *P* = 0.46; NDD-CKD group: MD: 0.30, 95%CI: -0.11–0.71, *P* = 0.16). The intra-group heterogeneity in the DD-CKD group is not significant (I^2^ = 28%,), while the heterogeneity between the two subgroups is significant (I^2^ = 67.1%). The results from the fixed-effects model were similar to the random-effects model (DD-CKD group: MD: -0.14, 95%CI: -0.41–0.13, *P* = 0.31; NDD-CKD group: MD:0.30, 95%CI: -0.11–0.71, *P* = 0.16; Fig. 3B). The heterogeneity between the two subgroups is also significant (I^2^ = 67.1%).

**Fig. 4:**
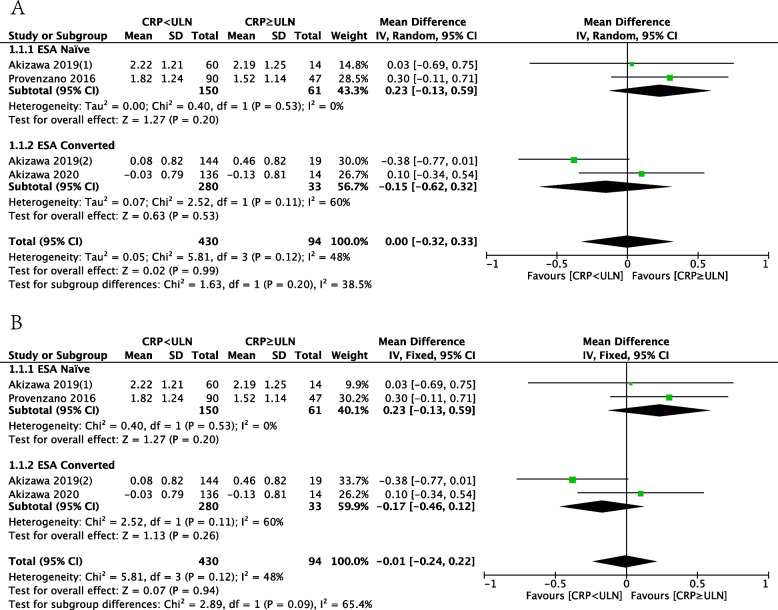
Forest plot of the effect of CRP levels on the efficacy of HIF-PHIs subgroup by previous ESA usage. **A** Random-effect model; **B** Fixed-effect model. CRP: c-reactive protein; ULN, upper limit of normal; ESA, erythropoiesis-stimulating agents; Mean, mean change of Hb from baseline to end of treatment, g/dl; SD, Standard deviation; CI: confidence interval; Total: Total number of patients in the study group; df: degrees of freedom; I^2^, I-squared

Subgroup analysis was also conducted based on prior ESA treatment. The ESA Naïve group included patients who had not receive ESA treatment previously, while the ESA-Converted group include those who had received ESA treatment before enrollment and switched to HIF-PHI treatment.. Each group comprised two studies. In the random-effects model, the results showed no difference in ∆Hb between patients with baseline CRP < ULN and CRP ≥ ULN in both the ESA Naïve and ESA Converted groups (ESA Naïve group: MD: 0.23, 95%CI: -0.13–0.59, *P* = 0.20; ESA Converted group: MD: -0.15, 95%CI: -0.62–0.32, *P* = 0.53; Fig. 4A). There was no intra-group heterogeneity in the ESA Naïve group (I^2^ = 0%), but significant intra-group heterogeneity in the ESA Converted group (I^2^ = 60%). The heterogeneity between the two subgroups was significant (I^2^ = 65.4%). The results from the random-effects model were similar to the fixed-effects model (ESA Naïve group: MD: 0.23, 95%CI: -0.13–0.59, *P* = 0.20; ESA Converted group: MD: -0.17, 95%CI: -0.46–0.12, *P* = 0.26; Fig. 4B), and heterogeneity between the two subgroups is not significant (I^2^ = 38.5%).

As all data were obtained from published literature, no subgroup analysis related to the data source was performed.

#### Sensitivity analysis

**Fig. 5:**
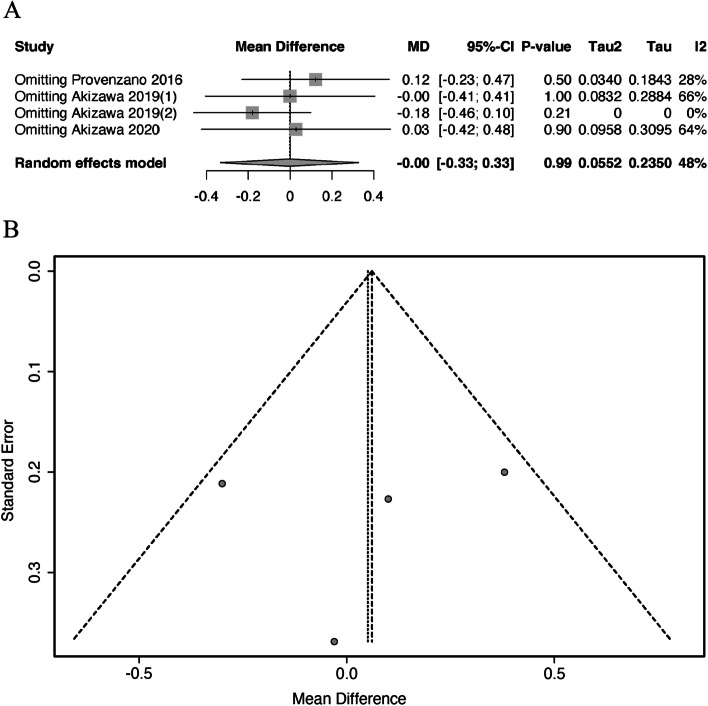
Sensitivity analysis and funnel plot of CRP levels on the efficacy of HIF-PHIs. **A** Sensitivity analyses by omitting studies individually. **B** Funnel plot MD, mean differences; CI, confidence intervals; I^2^, I-squared

Sensitivity analyses were employed by omitting studies individually, and the overall study effect ∆Hb was not statistically significant (95% CI all contain 0, Fig. 5A), consistent with the overall analysis. When analyzed after removing two studies by Provenzano et al*. *2016 and Akizawa et al. 2019 [2], the I^2^ statistic became zero.

#### Publication bias

Due to the limitation of the number of included studies, Begg and Egger tests could not be performed. Using a funnel plot for publication bias analysis (Fig. 5B), the studies were roughly distributed on either side of MD = 0.

### HIF-PHI drug efficacy in the CRP ≥ ULN group

#### Overall effect

**Fig. 6:**
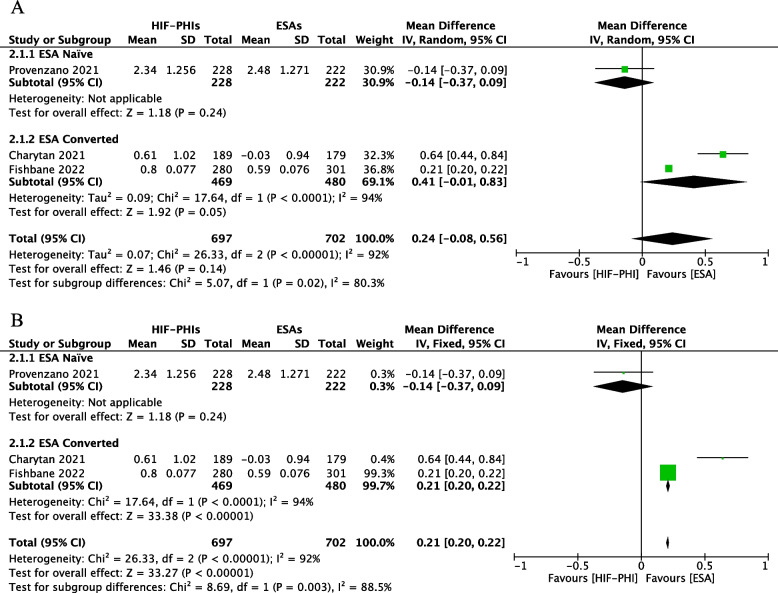
Forest plot of the effect of HIF-PHIs efficacy in the CRP ≥ ULN group subgroup by previous ESA usage. **A** Random-effect model; **B** Fixed-effect model. CRP: c-reactive protein; ULN, upper limit of normal; ESA, erythropoiesis-stimulating agents; Mean, mean change of Hb from baseline to end of treatment, g/dl; Total: Total number of patients in the study group; SD, Standard deviation; CI: confidence interval; df: degrees of freedom; I^2^, I-squared

Three studies reported ∆Hb from baseline to the EoT after treatment with HIF-PHI and ESA drugs in patients with CRP ≥ ULN at enrollment. The random-effects model analysis revealed no significant difference between the ESA group and the HIF-PHI group (MD: 0.24, 95%CI: -0.0.08–0.56, *P* = 0.14) (Fig. 6A). However, in the fixed-effects model analysis, the overall MD of ∆Hb in the ESA group was higher by 0.21 (95%CI: 0.20–0.22, *P* < 0.00001) (Fig. 6B) compared to the HIF-PHI group. Notably, overall heterogeneity was significant (I^2^ = 92%, *P* < 0.00001). According to the GRADE framework, the overall quality of evidence was very low for HIF-PHI drug efficacy in the CRP ≥ ULN group (Table 2).

### Subgroup analysis

Subgroup analysis based on previous ESA treatment included one study in the ESA Naïve group and two studies in the ESA-Converted group. In the random-effects model analysis, the MD of ∆Hb in the ESA group is higher by 0.41 (95%CI: -0.01–0.83, *P* < 0.00001; Fig. 6A) than in the HIF-PHI group. The intra-group difference heterogeneity was significant (I^2^ = 94%) and the heterogeneity between the two subgroups was also significant (I^2^ = 80.3%). In the fixed-effects model analysis of the ESA-Converted group, the MD of ∆Hb in the ESA group was higher by 0.21 (95%CI: 0.20–0.22, *P* < 0.00001; Fig. 6B) than that in the HIF-PHI group. The intra-group difference heterogeneity was significant (I^2^ = 94%) and the heterogeneity between the two subgroups was also significant (I^2^ = 88.5%).

No subgroup analysis was employed in this part based on whether the patients were dialysis-dependent or not, as both incident dialysis (ID-CKD) and dialysis-dependent patients(DD-CKD) represent end-stage renal diseases in the course of CKD and both essentially require dialysis to eliminate metabolic wastes from the body.

As the results of the study by Charytan et al*.* 2021 reported in the published literature were identical to those in the trial registration database, they were not extractable due to the presentation and no subgroup analysis was employed, as it was not considered as a potential source of reporting bias.

### Sensitivity analysis

**Fig. 7:**
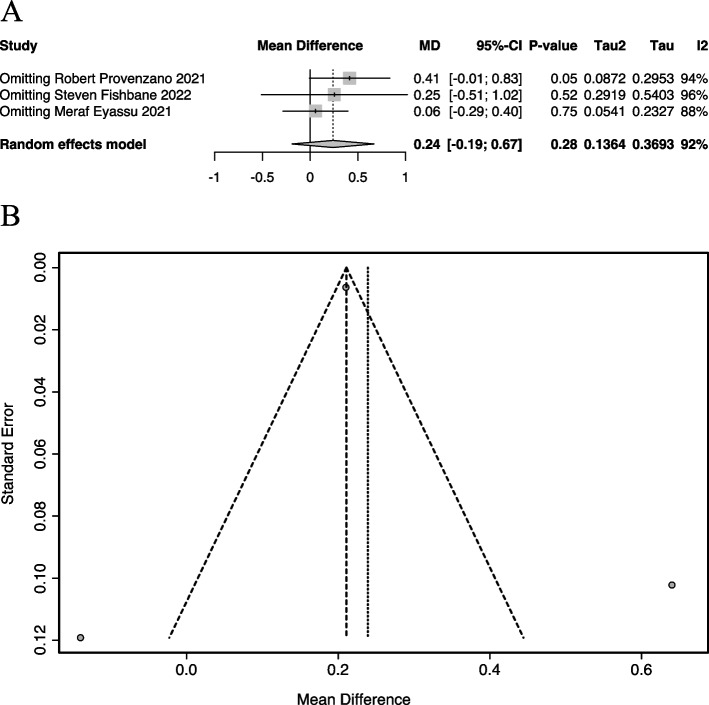
Sensitivity analysis and funnel plot of HIF-PHIs efficacy in the CRP ≥ ULN group. **A** Sensitivity analyses by omitting studies individually. **B** Funnel plot MD, mean differences; CI, confidence intervals; I^2^, I-squared

Sensitivity analyses were employed by omitting studies individually, and the overall study effect ∆Hb was not statistically significant (95%CI all contain 0, Fig. 7A), consistent with the overall analysis. The overall I^2^ statistic did not appear to change significantly with the omitting of a particular study.

### Publication bias

Due to the limitation of the number of included studies, Begg and Egger tests could not be performed. Publication bias analysis was conducted using funnel plots (Fig. 7B). Two studies fell on the outside of the confidence interval, considering a large heterogeneity between studies, which is consistent with the results of the heterogeneity test. Considering the similar sample sizes between the studies, the larger standard errors for these two studies contributed to the larger standard deviations.

### Comparison of HIF-PHI and ESA drug dosages

It is acknowledged that the ESA hyporesponsiveness in CKD patients, along with the escalation of drug dosage to achieve target hemoglobin concentrations, is associated with cardiovascular events, thrombosis, and mortality [24]. According to the Kidney Disease: Improving Global Outcomes (KDIGO) guidelines from 2012 [25], during maintenance therapy with ESA, a twofold increase in ESA dosage is required to maintain similar hemoglobin concentrations, and an increase of no more than 50% of its stable dose is considered indicative of ESA hyporesponsiveness. The ESA resistance index (ERI), which based on a ratio between ESA weekly dose per kilogram and hemoglobin concentration, is often used as a quantitative measure to assess ESA hyporesponsiveness [26]. However, current clinical studies on hypoxia-inducible factor prolyl hydroxylase inhibitors (HIF-PHIs) have not reported ERI, and the studies included in our analysis have not reported hemoglobin concentrations based on CRP levels. We summarized studies included in the analysis that reported drug dosages (Table 3) and calculated the drug dosages of ESA and roxadustat based on average weight. According to the conversion methods specified in the initial treatment protocols of ESA and rosuvastatin, a comparison of the approximate doses of the two drugs was made in patients from the CRP ≥ ULN group and CRP < ULN group during the baseline and throughout the study process. In patients treated with ESA in the group with CRP ≥ ULN, ESA dosage tended to increase over time, suggesting ESA hyporesponsiveness. In contrast, roxadustat dosage tended to decrease over time and was unrelated to CRP levels, indicating that inflammatory levels do not impact roxadustat efficacy. The converted roxadustat dosages were consistently lower than those of ESA, suggesting that it can exert its effects in a more efficient way.

**Table 3:** Comparison of HIF-PHI and ESA drug dosages^a^

Study	Interventions	Weight (kg)^2^	Dosage		Hemoglobin		Conclusion
			CRP < ULN	CRP ≥ ULN	CRP < ULN	CRP ≥ ULN	
Akizawa et al. 2020 [18]	Roxadustat	57.82 (11.97)	Baseline: 70 mg TIW	Baseline: 85 mg TIW	Baseline: 11 g/dL	Baseline: 10.9 g/L	Roxadustat dosage decreased over time and didn’t affect by CRP levels, whereas in an inflammatory state, Darbepoetin Alfa dosage increased over time, indicating ESA resistance. Roxadustat at 70 mg TIW is equivalent to Darbepoetin Alfa < 20ug/w^c^, in the CRP ≥ ULN group, ESA dosage is higher than roxadustat dosage during follow up
			During follow up: Consistently at 70 mg TIW before the 14th week, and declined to 50 mg TIW after the 14th week	During follow up: Increased to 100 mg TIW during the 1st and 4th to 7th weeks, decreased to 70 mg TIW after the 8th week, and then decreased to 50 mg TIW during the 13th and 20th to 23rd weeks	During follow up: Increased to 11.2 g/dL during the 1st to 4th weeks, fluctuated around 11 g/dL during the 6th to 24th weeks, mean ∆Hb_18-24w_^b^ -0.03(0.79)g/dL	During follow up: Increased to 11.3 g/dL during the 1st to 6th weeks, gradually decreased to 11 g/dL during the 7th to 16th weeks, mean ∆Hb_18-24w_^b^ -0.13 (0.81)g/dL	
	Darbepoetin Alfa	58.78 (12.90)	Baseline: 15ug/w	Baseline: 20 ug/w	Baseline: 11.1 g/dL	Baseline: 11.1 g/dL	
			During follow up: increased to 20 ug/w during the 4th to 18th weeks, declined to 15 ug/w after the 18th week	During follow up: Increased to 30ug/w after the 11th week	During follow up: Fluctuated around 11 g/dL, with slight decreasing trend, mean ∆Hb^b^ -0.03 (0.88) g/dL	During follow up: Fluctuated around 11 g/dL, with slight decreasing trend, mean ∆Hb^b^ -0.18 (0.94) g/dL	
Provenzano et al. 2021 [16]	Roxadustat	76.0 (18.5)	Baseline: 3.7 mg/kg/w	Baseline: 3.5 mg/kg/w	Baseline: 8.4 (1.0) g/dL^b^	Baseline: 8.4 (1.0) g/dL^b^	There was little difference in roxadustat drug dosage across different CRP groups, whereas the maximum Epoetin alfa drug dose was 30 IU/kg/w greater in the CRP ≥ ULN group than in the CRP < ULN group. Based on mean body weight, the roxadustat dose was less than 100 mg TIW and the Epoetin alfa dose was 76,700 IU/w. The two doses were in the same interval^d^, but roxadustat was closer to the lower limit of the interval and Epoetin alfa was closer to the upper limit of the interval
			During follow up: Gradually declined to 3 mg/kg/w at the 40th week	During follow up: Gradually declined to 3 mg/kg/w at the 44th week	During follow up: Mean ∆Hb increased to 2.6 g/dL from baseline to the 8th week, then fluctuated from 2.5 g/dL to 3 g/dL	During follow up: Mean ∆Hb increased to 2.4 g/dL from baseline to the 12th week, then fluctuated from 2.2 g/dL to 2.6 g/dL	
	Epoetin alfa	76.7 (19.1)	Baseline: 130 IU/kg/w	Baseline: 140 IU/kg/w	Baseline: 8.4 (1.0) g/dL^b^	Baseline: 8.4 (1.0) g/dL^b^	
			During follow up: Declined over time, fluctuated from 105 IU/kg/w to 130 IU/kg/w during the 8th to 52nd weeks	During follow up: Increased to 150 IU/kg/w at the 6th week, then decreased to 120 IU/kg/w at the 24th week, then fluctuated from 110 IU/kg/w to 130 IU/kg/w	During follow up: Mean ∆Hb increased to 2.6 g/dL from baseline to the 12th week, then fluctuated from 2.2 g/dL to 2.6 g/dL	During follow up: Mean ∆Hb increased to 2.6 g/dL from baseline to the 22nd week, then fluctuated from 2.4 g/dL to 2.6 g/dL	
Charytan et al. 2021 [17]	Roxadustat	84.3 (22.3)	Baseline: 4.4 mg/kg/w	Baseline: 4 mg/kg/w	Baseline: 10.30 (0.66) g/dL^b^	Baseline: 10.30 (0.66) g/dL^b^	Roxadustat dose remained stable during the follow up and was lower in the CRP ≥ ULN group than in the CRP < ULN group, while Epoetin alfa dose increased over time, indicating ESA resistance, with a more pronounced effect in inflammatory conditions. Calculated based on average weight, the roxadustat dosage is approximately 110 mg TIW, whereas the Epoetin alfa dosage is 86,600 IU/w. The ESA dosage is higher than that of roxadustat^c^
			During follow up: Remained stable, fluctuated from 4 mg/kg/w to 4.4 mg/kg/w	During follow up: Remained stable, fluctuated from 3.8 mg/kg/w to 4 mg/kg/w	During follow up: Mean ∆Hb increased to 0.75 g/dL from baseline to the 8th week, then gradually decreased to 0.1 g/dL at 48th week	During follow up: Mean ∆Hb increased to 0.8 g/dL from baseline to the 6th week, then gradually decreased to 0.4 g/dL at 48th week	
	Epoetin alfa	86.6 (23.0)	Baseline: 95 IU/kg/w	Baseline: 100 IU/kg/w	Baseline: 10.31 (0.66) g/dL^b^	Baseline: 10.31 (0.66) g/dL^b^	
			During follow up: Decreased to 80 IU/kg/w at the 6th week, then increased to 120 IU/kg/w at 44th week	During follow up: Gradually increased to 180 IU/kg/w at 48th week	During follow up: Mean ∆Hb increased to 0.2 g/dL at 1st week, decreased to -0.1 g/dL at 14th week, fluctuated from 0 g/dL to -0.2 g/dL during the 14th to 52nd weeks	During follow up: Mean ∆Hb fluctuated from 0.1 g/dL to -0.2 g/dL	

^a^Data not labeled in the original article are roughly estimated based on the data presented in the image

^b^Mean ± standard deviation

^c^Equivalent doses are converted as given in the study protocol

^d^Equivalent doses are converted according to the conversions given by other studies that used Epoetin alfa as control

## Discussion

### Principal findings

The results of this systematic review and meta-analysis indicate that HIF-PHIs demonstrate comparable efficacy across various CRP levels, irrespective of dialysis dependence and prior ESA treatment. In the CRP ≥ ULN group, the efficacy of HIF-PHIs was found to be akin to standard ESA treatment. It’s worth noting that the fixed-effects model and random-effects model produced differing results due to the significant heterogeneity among the included studies. Prior systematic review on the HIF-PHI drugs have primarily focused on safety and efficacy, with no prior studies delving into the impact of CRP levels on HIF-PHI efficiency. While extracting data from the literature, we encountered several articles indicating no significant differences in the effects of HIF-PHIs between the CRP < ULN and CRP ≥ ULN groups, yet we were unable to retrieve extractable data [17, 27–35]. In a study by Chenet al*.*, EoT hemoglobin concentrations were comparable in the CRP ≥ ULN and CRP < ULN groups among patients treated with similar doses of roxadustat. Conversely, in patients treated with ESA, those in the CRP ≥ ULN group received higher doses of ESA, but their mean hemoglobin remained lower than in the CRP < ULN group [35]. Data from Hou et al. and Charytanet al*.* demonstrated that CRP levels did not influence the efficacy of roxadustat [17, 27], and similar findings were reported in a study on another HIF-PHI preparation, Molidustat [32]. Two publications assessing the efficacy of HIF-PHI versus placebo in the CRP ≥ ULN group both indicated the efficacy of HIF-PHI in increasing hemoglobin concentrations compared to placebo. However, due to differing outcome parameters (Fishbane et al. 2021 [36] used Adjusted LSM and Coyne et al. 2021 [37] used MD), they were ultimately excluded from the study and unavailable for pooled analysis. The qualitative results reported in these studies align with the findings of our meta-analysis. We attempted to reach out to the authors for additional data, but no new information became available. A comparative analysis of drug dosages between ESA and roxadustat reveals a consistent observation of ESA hyporesponsiveness across various studies, particularly evident in the CRP ≥ ULN group. In contrast, roxadustat consistently maintains its efficacy.

### Implications from subgroup analyses

In the comparison between the CRP ≥ ULN group and the CRP < ULN group among patients treated with HIF-PHI, subgroup analysis based on dialysis dependence revealed low intra-group heterogeneity and high between-group heterogeneity. This indicates that dialysis is a significant factor influencing the heterogeneity of the included studies. Subgroup analyses based on previous antianemia treatment showed that the random-effects model had lower inter-group heterogeneity than the fixed-effects model. However, the substantial intra-group heterogeneity observed in the ESA conversion group suggests that grouping based on previous antianemia treatment did not fully resolve the differences in the included studies.

In the analysis of the efficacy of HIF-PHIs in the CRP ≥ ULN group, subgroup analysis based on previous antianemia treatment revealed considerable intra- and inter-group heterogeneity. As such, we believe that this subgroup analysis may not effectively elucidate the source of heterogeneity. It is evident that the 95% confidence interval of Fishbaneet al*.* 2022 is narrow, weighting it highly in the overall results. Given the absence of a significant difference in disease stage between IDD-CKD and DD-CKD, a subgroup analysis was not conducted between them. Due to the limited number of included studies, additional subgroup analyses were not attempted. Consequently, we are cautious about drawing definitive conclusions based on the studies included in the analysis thus far.

### Implications for clinical and research

In CKD, anemia primarily result from a relative deficiency of EPO, with additional factors such as uremic toxins, chronic inflammation, impaired iron metabolism, and shortened erythrocyte lifespan contributing to its complexity [5, 8, 9]. While recombinant human EPO has been a standard treatment for CKD-related anemia for over two decades, some studies have indicated that targeting higher hemoglobin concentrations with EPO treatment or higher ESA doses may elevate the risk of stroke, hypertension, thrombosis, and mortality [10, 24]. Following the elucidation of mechanisms related to the role of HIFs as oxygen receptors exerting transcriptional regulation [38–40], HIF-PHIs have been developed as small-molecule drugs that stabilize HIFs. HIF-PHIs can exert their antianemia effects through multiple mechanisms, with the most direct approach being the stabilization of HIF-2 to promote EPO production [38, 41]. In addition, the upregulation of transferrin, cephalin, and transferrin receptor 1 by HIF-1 facilitates iron transport, while the upregulation of duodenal cytochrome B (DcytB) and divalent metal transporter 1 (DMT1) expression by HIF-2 enhances intestinal iron absorption [3]. Both pathways contribute to an increase in the material available for hemoglobin synthesis, enabling HIF-PHIs to correct anemia in a more efficient manner. Furthermore, a systematic review has demonstrated that HIF-PHIs significantly reduce hepcidin levels compared to placebo [42]. HIF-PHIs have also exhibited a more substantial reduction in hepcidin levels in certain clinical studies when compared to standard care with ESA [15, 16]. Hepcidin is considered a key molecule contributing to inflammatory anemia. In previous practice involving ESA treatment for CKD-related anemia, elevated inflammation levels were often associated with ESA hyporesponsiveness [8, 9]. By diminishing hepcidin levels, HIF-PHIs can enhance iron utilization for hemoglobin synthesis. Sugahara et al*.* suggest that one of the advantages of HIF-PHIs is their efficacy in ESA hyporesponsive patients [3].

Previous systematic reviews of HIF-PHIs have predominantly focused on the drugs’ efficacy and safety, with some studies delving into the impact of HIF-PHIs on lipid metabolism and iron metabolism [42, 43]. Few systematic reviews have specifically addressed the relationship between inflammation and the efficacy of HIF-PHIs. Our original intent in conducting this systematic review was to explore the effect of inflammation on HIF-PHIs efficacy and, consequently, the potential application of HIF-PHIs in treating inflammatory anemia. Inflammatory anemia stands as the second most common cause of anemia, following iron deficiency anemia [4]. Current treatment for inflammatory anemia include blood transfusion, iron supplementation, and ESA therapy. ESA can address inflammatory anemia by reducing inflammation through inhibiting proinflammatory immune pathways, lowering hepcidin levels, and promoting erythropoiesis [44–46]. However, the existing treatments fall short due to limitations in efficacy and side effects [4]. Some patients with inflammatory anemia exhibit poor responsiveness to ESA therapy, and higher hemoglobin treatment targets with ESA therapy are linked to an elevated risk of stroke, hypertension and thrombosis [10]. Several drugs targeting iron-regulated elements and their related pathways are currently in development [47]. Based on the mechanism of action of the drugs and existing clinical data, we believe that HIF-PHIs currently hold the potential to effectively treat inflammatory anemia. On the one hand, HIF-PHIs have demonstrated the ability to exert anti-anemic effects under inflammatory conditions, as explored in this article. Moreover, this perspective finds support in animal models. In a rat model of inflammatory anemia, roxadustat corrected anemia by reducing hepcidin expression in the liver and increasing the expression of two genes involved in intestinal iron absorption (DcytB and DMT1) [48]. Importantly, it did not alter the level of inflammation in rats, suggesting that its role in correcting anemia operates downstream of inflammation. HIF-PHIs can also promote EPO production by directly enhancing hematopoiesis and improving hematopoietic response in the inflammatory state to effectively treat inflammatory anemia [44–46]. Our study demonstrates that the efficacy of roxadustat remains unaffected by inflammation. It effectively maintains hemoglobin concentrations even in the presence of inflammatory conditions, without exhibiting the hyporesponsiveness commonly associated with ESA. Additionally, a meta-analysis of HIF-PHI drugs on iron metabolism revealed that [49], compared to ESA, HIF-PHI drugs can elevate levels of iron, total iron-binding capacity, and transferrin while reducing hepcidin levels and the dosage of intravenous iron preparations. This is particularly crucial for treating inflammatory anemia characterized by impaired iron utilization.

Although the prospect of stabilizing HIF to treat inflammatory anemia appears promising, the complex role of HIF in inflammatory diseases complicates this scenario. It remains to be seen whether HIF-PHIs can ameliorate anemia associated with various inflammatory diseases, such as rheumatoid arthritis and inflammatory bowel disease, without exacerbating inflammatory activity and tissue damage. Documentation indicate that HIF can contribute to the development of these diseases. In inflammatory bowel disease, HIF expression in Th17 cells, pivotal mediators of IBD, adversely affects the progression of Crohn's disease [50]. Similarly, in rheumatoid arthritis, HIF-1a perpetuates the development of RA by activating pathways involved in synovial inflammation, angiogenesis, cartilage destruction, and bone erosion [51–54]. HIF-dependent genes play roles in tumor angiogenesis, cell survival, tumor metastasis, and invasion, and are associated with immunosuppressive effects on tumor cells [55]. Given HIF’s role as an oxygen-sensing mechanism for cells and the broad diversity of metabolic pathways regulated by HIF, additional considerations regarding the safety of HIF-PHIs arise. Therefore, despite the demonstrated effectiveness of HIF-PHIs in CKD patients with high inflammatory status, further mechanistic and clinical studies are essential to determine whether HIF-PHIs can be applied successfully to treat anemia caused by inflammatory diseases.

### Limitations

Our study is subject to certain limitations. Firstly, being a systematic review, the quality of the included studies inevitably influences our findings. The studies incorporated into our analysis are susceptible to biases arising from issues such as blinding during implementation, loss to follow-up, and corporate sponsorship, all of which impact the robustness of our conclusions. Secondly, the reliability of the results is affected by variations in the design of the included studies, the setting of ULN values, the choice of CRP measures, the stage of the patients' disease, and some unexplained heterogeneity evident from our subgroup analysis. Thirdly, the relative variability of Hb levels varies considerably, and in our initial meta-analysis, three of the four studies were derived from the same consortium, potentially influencing the overall significance of the results due to the study population’s specific genetic background and potential presence of a distinct treatment algorithm. Consequently, our findings may not be universally applicable and may differ from in global regions. In addition, from the perspective of completing the evidence chain, none of the current studies enrolled patients with severe inflammation or under-response to ESA. Finally, employing the GRADE framework revealed that the low or very low quality evidence in study outcomes is largely attributable to study design and inconsistency. We anticipate that future studies of higher quality will contribute more robust evidence to address the lingering questions.

## Conclusions

Our systematic review reveals that roxadustat demonstrates consistent efficacy across different CRP levels. Particularly noteworthy is its ability to maintain efficacy comparable to ESA in the CRP ≥ ULN group without the need for dose escalation. This finding has implications for advancing research focused on the efficacy of HIF-PHI in patients with CKD experiencing an inflammatory state.

### Supplementary Information

**Additional file 1.**

## Data Availability

All data generated or analyzed during this study are included in this published article and its supplementary information files.

## Abbreviations

CI: Confidence intervals
CKD: Chronic kidney disease
CRP: C-reactive protein
DcytB: Duodenal cytochrome B
DD-CKD: Dialysis-dependent CKD
DMT1: Divalent metal transporter 1
∆Hb: Hemoglobin concentration changes
EoT: The end-of-treatment
EPO: Erythropoietin
ESA: Erythropoiesis-stimulating agents; Evaluation framework
GRADE: The Grading of Recommendations Assessment, Development, and Evaluation (GRADE) framework
HIF: Hypoxia-inducible factor
HIF-PHIs: Hypoxia-inducible factor-prolyl hydroxylase inhibitors
ICTRP: The International Clinical Trials Registry Platform
MD: Mean differences
NDD-CKD group: Non-dialysis-dependent CKD
PRISMA: The Preferred Reporting Items for Systematic Evaluation and Meta-Analysis
RCT: Randomized-controlled trials
RevMan: Cochrane's Review Manager
ULN: Upper limit of normal
