# Supplementary material for: Impact of C-reactive protein on the effect of Roxadustat for the treatment of anemia in chronic kidney disease: a systematic review of randomized controlled trials
Source: BMC Nephrol. 2024 Feb 5;25:47. doi: 10.1186/s12882-024-03474-5 (PMC10840261; doi:10.1186/s12882-024-03474-5)
Supplement: Supplementary file 1 — Additional file 1. [file 12882_2024_3474_MOESM1_ESM.docx]

**Supplementary materials**

**Search strategy**

Pubmed

| No. | Subjects | Items |
| --- | --- | --- |
| #1 | CKD | "Renal Insufficiency, Chronic"[MeSH Terms] |
| #2 |  | Chronic Renal Insufficiencies"[Title/Abstract] |
| #3 |  | "Chronic Renal Insufficiency"[Title/Abstract] |
| #4 |  | "Kidney Insufficiency, Chronic"[Title/Abstract] |
| #5 |  | "Chronic Kidney Insufficiency"[Title/Abstract] |
| #6 |  | "Chronic Kidney Diseases"[Title/Abstract] |
| #7 |  | "Chronic Kidney Disease"[Title/Abstract] |
| #8 |  | "Disease, Chronic Kidney"[Title/Abstract] |
| #9 |  | "Diseases, Chronic Kidney"[Title/Abstract] |
| #10 |  | "Kidney Disease, Chronic"[Title/Abstract] |
| #11 |  | "Kidney Diseases, Chronic"[Title/Abstract] |
| #12 |  | "Chronic Renal Diseases"[Title/Abstract] |
| #13 |  | "Chronic Renal Disease"[Title/Abstract] |
| #14 |  | "Disease, Chronic Renal"[Title/Abstract] |
| #15 |  | "Diseases, Chronic Renal"[Title/Abstract] |
| #16 |  | "Renal Disease, Chronic"[Title/Abstract] |
| #17 |  | "Renal Diseases, Chronic"[Title/Abstract] |
| #18 |  | "CKD"[Title/Abstract] |
| #19 |  | #1 OR #2 OR #3 OR #4 OR #5 OR #6 OR #7 OR #8 OR #9 OR #10 OR #11 OR #12 OR #13 OR #14 OR #15 OR #16 OR #17 OR #18 OR #19 |
| #20 | HIF-PHI | "HIF-PHI"[Title/Abstract] |
| #21 |  | "hypoxia-inducible factor-prolyl hydroxylase inhibitors"[Title/Abstract] |
| #22 |  | "roxadustat"[Title/Abstract] |
| #23 |  | "FG-4592"[Title/Abstract] |
| #24 |  | "FG4592"[Title/Abstract] |
| #25 |  | "GSK1278863" [Title/Abstract] |
| #26 |  | "daprodustat"[Title/Abstract] |
| #27 |  | "desidustat"[Title/Abstract] |
| #28 |  | "enarodustat"[Title/Abstract] |
| #29 |  | "JTZ-951"[Title/Abstract] |
| #30 |  | "molidustat"[Title/Abstract] |
| #31 |  | "BAY 85-3934"[Title/Abstract] |
| #32 |  | "vadadustat"[Title/Abstract] |
| #33 |  | "AKB-6548"[Title/Abstract] |
| #34 |  | "HIF-PHIs"[Title/Abstract] |
| #35 |  | "hypoxia-inducible factor-prolyl hydroxylase inhibitor"[Title/Abstract] |
| #36 |  | #20 OR #21 OR #22 OR #23 OR #24 OR #25 OR #26 OR #27 OR #28 OR #29 OR #30 OR #31 OR #32 OR #33 OR #34 OR #35 |
| #37 | RCT | clinical[tiab] |
| #38 |  | trial[tiab] |
| #39 |  | "clinical trials as topic"[mesh] |
| #40 |  | "clinical trial"[pt] |
| #41 |  | random*[tiab] |
| #42 |  | "random allocation"[mesh] |
| #43 |  | "therapeutic use"[sh] |
| #44 |  | (#37 AND #38) OR #39 OR #40 OR #41 OR #42 OR #43 |
| #45 | Search strategy | #19 AND #36 AND #44 |

Cochrane

| No. | Search |
| --- | --- |
| #1 | MeSH descriptor: [Renal Insufficiency, Chronic] explode all trees |
| #2 | (Chronic Kidney Disease):ti,ab,kw OR (Diseases, Chronic Kidney):ti,ab,kw OR (Renal Diseases, Chronic):ti,ab,kw OR (Kidney Disease, Chronic):ti,ab,kw OR (Chronic Renal Disease):ti,ab,kw (Word variations have been searched) |
| #4 | (Kidney Diseases, Chronic):ti,ab,kw OR (Diseases, Chronic Renal):ti,ab,kw OR (Chronic Renal Diseases):ti,ab,kw OR (Chronic Kidney Diseases):ti,ab,kw OR (Disease, Chronic Kidney):ti,ab,kw (Word variations have been searched) |
| #5 | (Renal Insufficiencies, Chronic):ti,ab,kw OR (Chronic Renal Insufficiency):ti,ab,kw OR (Kidney Insufficiency, Chronic):ti,ab,kw OR (Chronic Kidney Insufficiencies):ti,ab,kw (Word variations have been searched) |
| #6 | #1 OR #2 OR #3 OR #4 OR #5 |
| #7 | (roxadustat):ti,ab,kw OR (FG-4592):ti,ab,kw OR (FG4592):ti,ab,kw AND (GSK1278863):ti,ab,kw (Word variations have been searched) |
| #8 | (daprodustat):ti,ab,kw (Word variations have been searched) |
| #9 | (desidustat):ti,ab,kw (Word variations have been searched) |
| #10 | (enarodustat):ti,ab,kw OR (JTZ-951):ti,ab,kw (Word variations have been searched) |
| #11 | (vadadustat):ti,ab,kw AND (AKB-6548):ti,ab,kw (Word variations have been searched) |
| #12 | (molidustat):ti,ab,kw (Word variations have been searched) |
| #13 | (HIF-PHI):ti,ab,kw OR (HIF-PHIs):ti,ab,kw OR (hypoxia-inducible factor-prolyl hydroxylase inhibitor):ti,ab,kw OR (hypoxia-inducible factor-prolyl hydroxylase inhibitors):ti,ab,kw (Word variations have been searched) |
| #14 | #7 OR #8 OR #9 OR #10 OR #11 OR #12 OR #13 |
| #15 | #6 AND #14 |

Embase

| No. | Query |
| --- | --- |
| #1 | 'chronic kidney failure'/exp |
| #2 | 'chronic kidney failure':ti,ab,kw OR 'chronic kidney disease':ti,ab,kw OR 'chronic kidney disorder':ti,ab,kw OR 'chronic kidney insufficiency':ti,ab,kw OR 'chronic nephropathy':ti,ab,kw OR 'chronic renal disease':ti,ab,kw OR 'chronic renal failure':ti,ab,kw OR 'chronic renal insufficiency':ti,ab,kw OR 'kidney chronic failure':ti,ab,kw OR 'kidney disease, chronic':ti,ab,kw OR 'kidney failure, chronic':ti,ab,kw OR 'kidney function, chronic disease':ti,ab,kw OR 'renal insufficiency, chronic':ti,ab,kw |
| #3 | #1 OR #2 |
| #4 | 'hypoxia inducible factor prolyl hydroxylase inhibitor'/exp |
| #5 | 'hypoxia inducible factor prolyl hydroxylase inhibitor':ti,ab,kw OR 'hif proline hydroxylase inhibitor':ti,ab,kw OR 'hif prolyl hydroxylase inhibitor':ti,ab,kw OR 'hypoxia inducible factor proline dioxygenase inhibitor':ti,ab,kw OR 'hypoxia inducible factor proline hydroxylase inhibitor':ti,ab,kw |
| #6 | #4 OR #5 |
| #7 | 'roxadustat'/exp |
| #8 | roxadustat:ti,ab,kw OR (2:ti,ab,kw AND '4 hydroxy 1 methyl 7 phenoxy 3 isoquinolinecarboxamido':ti,ab,kw AND 'acetic acid':ti,ab,kw) OR ('2 [':ti,ab,kw AND '4 hydroxy 1 methyl 7 phenoxyisoquinoline 3 carbonyl':ti,ab,kw AND 'amino] acetic acid':ti,ab,kw) OR 'asp 1517':ti,ab,kw OR asp1517:ti,ab,kw OR 'azd 9941':ti,ab,kw OR azd9941:ti,ab,kw OR evrenzo:ti,ab,kw OR 'fg 4592':ti,ab,kw OR fg4592:ti,ab,kw OR ('n [':ti,ab,kw AND '4 hydroxy 1 methyl 7 phenoxy 3 isoquinolinyl':ti,ab,kw AND 'carbonyl] glycine':ti,ab,kw) OR ('n [':ti,ab,kw AND '4 hydroxy 1 methyl 7 phenoxyisoquinolin 3 yl':ti,ab,kw AND 'carbonyl] glycine':ti,ab,kw) |
| #9 | #7 OR #8 |
| #10 | 'molidustat'/exp |
| #11 | molidustat:ti,ab,kw OR (1:ti,ab,kw AND '6 morpholinopyrimidin 4 yl':ti,ab,kw AND 4:ti,ab,kw AND '1h 1, 2, 3 triazol 1 yl':ti,ab,kw AND '1h pyrazol 5 ol':ti,ab,kw) OR ('1, 2 dihydro 2':ti,ab,kw AND '6 morpholino 4 pyrimidinyl':ti,ab,kw AND 4:ti,ab,kw AND '1h 1, 2, 3 triazol 1 yl':ti,ab,kw AND '3h pyrazol 3 one':ti,ab,kw) OR ('1, 2 dihydro 2 [6':ti,ab,kw AND '4 morpholinyl':ti,ab,kw AND '4 pyrimidinyl] 4':ti,ab,kw AND '1h 1, 2, 3 triazol 1 yl':ti,ab,kw AND '3h pyrazol 3 one':ti,ab,kw) OR ('2 [6':ti,ab,kw AND '4 morpholinyl':ti,ab,kw AND '4 pyrimidinyl] 4':ti,ab,kw AND '1h 1, 2, 3 triazol 1 yl':ti,ab,kw AND '1, 2 dihydro 3h pyrazol 3 one':ti,ab,kw) OR ('2 [6':ti,ab,kw AND 'morpholin 4 yl':ti,ab,kw AND 'pyrimidin 4 yl] 4':ti,ab,kw AND '1h 1, 2, 3 triazol 1 yl':ti,ab,kw AND '1, 2 dihydro 3h pyrazol 3 one':ti,ab,kw) OR 'bay 1053048':ti,ab,kw OR 'bay 85 3934':ti,ab,kw OR 'bay 85-3934':ti,ab,kw OR 'bay1053048':ti,ab,kw OR 'bay85 3934':ti,ab,kw OR 'bay85-3934':ti,ab,kw OR 'molidustat sodium':ti,ab,kw OR 'musredo':ti,ab,kw |
| #12 | #10 OR #11 |
| #13 | 'daprodustat'/exp |
| #14 | daprodusta:ti,ab,kw OR (2:ti,ab,kw AND '1, 3 dicyclohexyl 2, 4, 6 trioxohexahydropyrimidine 5 carboxamido':ti,ab,kw AND 'acetic acid':ti,ab,kw) OR 'gsk 1278863':ti,ab,kw OR 'gsk 1278863a':ti,ab,kw OR 'gsk1278863':ti,ab,kw OR 'gsk1278863a':ti,ab,kw OR 'bay 1053048':ti,ab,kw OR 'bay 85 3934':ti,ab,kw OR ('n [':ti,ab,kw AND '1, 3 dicyclohexylhexahydro 2, 4, 6 trioxo 5 pyrimidinyl':ti,ab,kw AND 'carbonyl] glycine':ti,ab,kw) OR ('n [':ti,ab,kw AND '1, 3 dicyclohexylhexahydro 2, 4, 6 trioxopyrimidin 5 yl':ti,ab,kw AND 'carbonyl] glycine':ti,ab,kw) |
| #15 | #13 OR #14 |
| #16 | 'vadadustat'/exp |
| #17 | vadadustat:ti,ab,kw OR 'akb 6548':ti,ab,kw OR akb6548:ti,ab,kw OR ('n [5':ti,ab,kw AND '3 chlorophenyl':ti,ab,kw AND '3 hydroxypyridine 2 carbonyl] glycine':ti,ab,kw) OR ('n [ [5':ti,ab,kw AND '3 chlorophenyl':ti,ab,kw AND '3 hydroxy 2 pyridinyl] carbonyl] glycine':ti,ab,kw) OR 'pg 1016548':ti,ab,kw OR pg1016548:ti,ab,kw |
| #18 | #16 OR #17 |
| #19 | 'desidustat'/exp |
| #20 | desidustat:ti,ab,kw OR ('n [1':ti,ab,kw AND cyclopropylmethoxy:ti,ab,kw AND '4 hydroxy 2 oxo 1, 2 dihydroquinoline 3 carbonyl] glycine':ti,ab,kw) |
| #21 | #19 OR #20 |
| #22 | 'enarodustat'/exp |
| #23 | enarodustat:ti,ab,kw OR ('n [7 hydroxy 5':ti,ab,kw AND '2 phenylethyl':ti,ab,kw AND '[1, 2, 4] triazolo [1, 5 a] pyridine 8 carbonyl] glycine':ti,ab,kw) |
| #24 | #22 OR #23 |
| #25 | #6 OR #9 OR #12 OR #15 OR #18 OR #21 OR #24 |
| #26 | 'clinical':ti,ab AND 'trial':ti,ab OR 'clinical trial'/exp OR random* OR 'drug therapy':lnk |
| #27 | #3 AND #25 AND #26 |
